# *Origanum dubium* (Cypriot Oregano) as a Promising Sanitizing Agent against *Salmonella enterica* and *Listeria monocytogenes* on Tomato and Cucumber Fruits

**DOI:** 10.3390/biology11121772

**Published:** 2022-12-06

**Authors:** Panayiota Xylia, Antonios Chrysargyris, Panagiota Miltiadous, Nikolaos Tzortzakis

**Affiliations:** 1 Department of Agricultural Sciences, Biotechnology and Food Science, Cyprus University of Technology, Limassol 3036, Cyprus; 2 Department of Nursing, Faculty of Health Sciences, Cyprus University of Technology, Limassol 3036, Cyprus

**Keywords:** cucumber, fresh produce quality, food safety, *Origanum dubium*, sanitizing agents, tomato

## Abstract

**Simple Summary:**

Tomato and cucumber are two of the most important and frequently produced and consumed fresh produce. Both of them are highly perishable with various postharvest sanitation techniques to be applied that reduce postharvest losses and extend the shelf life of vegetables and fruits. Chemical sanitizers are limited in the postharvest industry and are subjected to consumer constraints. Essential oils derived from medicinal and aromatic plants are attracting increased interest as natural sanitizers due to their well-known antimicrobial and antioxidant properties.

**Abstract:**

In recent years, the use of natural products such as essential oils (EOs) and other plant extracts for the preservation of fresh produce has attracted much interest from the food industry. Many endemic medicinal and aromatic plants, such as Cypriot oregano (*Origanum dubium*), present a plethora of properties that can be utilized by the fruit and vegetable sectors of the food industry. The purpose of the present study was to assess the effects of *O. dubium* EO and hydrosol (at different concentrations and durations of dipping application) for the preservation of tomato and cucumber fruit quality, and their effectiveness as sanitizing agents against two foodborne pathogens (*Listeria monocytogenes* and *Salmonella enterica*). The results of this study indicated that increased concentrations of EO, combined with a longer duration of application, resulted in less marketable fruit compared to hydrosol application. Interestingly, EO application at lower concentrations and shorter durations of application (i.e., 0.01% for 5 min) increased fruit antioxidant, ascorbic acid and carotenoid levels (for tomato fruit), suggesting an increase in the nutritional value of the treated fruit, compared to the control. EO and hydrosol were able to decrease the bacterial populations (both bacteria) on fruits. Both products were especially effective against *L. monocytogenes*, even seven days after their application and storage at 11 °C (up to an approx. 3 log reduction with the EO application). Overall, the results of this study suggest that the use of *O. dubium* EO and hydrosol could be considered as alternative sanitation means for tomatoes and cucumbers.

## 1. Introduction

Interest in the investigation of unexploited, indigenous medicinal and aromatic plants (MAPs) and their properties is currently increasing [1,2,3]. Cypriot oregano (*Origanum dubium* Boiss.) is a perennial shrub of the *Origanum* genus belonging to the Lamiaceae family, and grows around the Mediterranean area. This plant, as well as its essential oil, infusion and other forms, has been used as remedy for many centuries due to its antioxidant and antiseptic/antimicrobial (i.e., antibacterial, antifungal) properties [4,5,6,7]. In Cyprus, *O. dubium* has been used as an appetizer and a curative means for many health problems such as gastrointestinal ailments (i.e., diarrhea, stomach ache, dyspepsia, and intestinal, liver and gall disorders), influenza, headache, toothache and respiration ailments (i.e., asthma, cough, common cold and bronchitis) [5,7]. Moreover, due to its unique and spicy fragrance, *O. dubium* has also been used in aromatherapy, perfumery and the food industry (meat products, bakeries, condiments and beverages) [4]. *Origanum dubium* has only recently been commercially cultivated by farmers in Cyprus, and approximately 20 ha are planned annually in open fields, with an estimated total production of 50 tons of dried product [8]. However, the sustainable agricultural practices in its cultivation are unknown (i.e., water and fertilizer management).

The increased need for and consumption of fresh produce as a part of a healthy lifestyle have been linked with increased numbers of foodborne illnesses [9]. Outbreaks regarding the presence of foodborne pathogens have been announced throughout the years implicating the consumption of fresh produce as well as minimally processed fruits and vegetables. According to the European Centre for Disease Prevention and Control (ECDC), salmonellosis and listeriosis are the second and fifth most commonly reported foodborne infections in humans after campylobacteriosis, respectively [10].

In recent years, there has been a turn towards the investigation of natural products as alternative sanitizing agents in fresh produce washing steps. This need derived from consumers’ demands for decreased use of synthetic chemical substances during food processing and preparation [11,12]. Moreover, the food industry searches for alternative means in order to eliminate the use of chlorine and chlorine-based products, since they present harm to human health and the environment [13,14]. Natural extracts such as essential oils (EOs) and hydrosols from aromatic plants are generally recognized as safe (GRAS) to use in the food industry and their use in food is also under EC Regulation No. 1334/2008 on flavorings and certain food ingredients with flavoring properties for use in and on foods [15,16].

Many EOs (i.e., sage, Greek oregano, eucalyptus and rosemary) and other natural extracts have been previously used for the preservation of fresh produce and minimally processed fruits and vegetables with encouraging results [1,17,18,19,20,21,22,23]. Essential oils at high concentrations might cause phytotoxicity and oxidative stress on fresh produce, as was evidenced in vaporized tomato fruit with sage EO [17]. Essential oils’ biocidal activity is not only related to the main component of the oil but also to the synergistic action of the components at a relevantly high percentage in the oil [24]. In addition, another by-product derived from EO extraction/distillation is the hydrosol from MAPs, which also presents significant antimicrobial and antioxidant properties, and some applications on fresh produce have already been reported [11,25,26,27,28,29,30]. Their hydrosol biological activity (i.e., antimicrobial properties) is attributed to their composition, and especially to the main and secondary constituents, which, on occasion, are similar to or completely different from their EO [15].

Cypriot oregano EO has been shown in previous reports to exhibit antimicrobial and antioxidant activities [4,7]. Previous studies have shown the antimicrobial activity of *O. dubium* EO against a plethora of pathogenic bacteria including *Escherichia coli*, *Pseudomonas aeruginosa*, *Staphylococcus aureus*, *Salmonella* Enteritidis, as well as the opportunistic pathogenic yeast *Candida albicans* [4,31]. However, there is no reference in its application on fresh produce preservation and in general as a postharvest sanitizer under storage conditions. The present study aimed to evaluate (i) the effects of *O. dubium* EO and hydrosol on tomato and cucumber fruit quality attributes as well as (ii) the efficacy of these products against two main foodborne pathogens (*Salmonella enterica* and *Listeria monocytogenes*) inoculated on those fruits during storage.

## 2. Materials and Methods

### 2.1. Plant Material

Fresh tomato (*Solanum lycopersicum* cv. Torry F1) and cucumber (*Cucumis sativus* cv. PS-64) fruits were obtained from a local producer (Limassol, Cyprus). Fresh produce was homogenously selected so that fruits presented uniformity in size and appearance, with no physical defects or injuries. After their transfer to the laboratory, the fruits were washed with chlorinated water (using 0.05% *v*/*v* sodium hypochlorite-NaOCl), and then, rinsed three times with sterile water, in order to remove/eliminate their native microflora.

Fresh Cypriot oregano (*Origanum dubium*) plants were collected from Cyprus University of Technology’s experimental farm/greenhouse, where they were grown in soil. The plants used in the present study originated from the Cypriot National Center of Aromatic Plants (Nicosia, Cyprus). The plant material was dried in an oven (air-dried at 42 °C). The EO was obtained via hydrodistillation using Clevenger apparatus for 3 h, and stored in umber glass bottles at −20 °C until use. The hydrosol (water remaining after hydrodistillation) was also collected, filtered (using cheese cloth for removing any solid residues) and stored at 4 °C until use. The composition of *O. dubium* EO was determined via Gas Chromatography–Mass Spectrometry (GC/MS; Shimadzu GC2010 gas chromatograph-interfaced Shimadzu GC/MS QP2010 plus mass spectrometer, Kyoto, Japan) according to Chrysargyris et al. [32], with the main constituents being carvacrol (averaged at 70.4%), p-cymene (averaged at 4.8%) and γ-terpinene (averaged at 3.4%), as reported previously [4].

### 2.2. Preliminary Screening

Preliminary screening was performed as described below for the determination of optimum doses (combination of concentration and duration of application) for each commodity that did not negatively affect their quality. After washing with chlorinated water (0.05% NaOCl), the fruits were air-dried and labeled. The fruits were dipped in treatment solutions of different EO or hydrosol concentrations (0.01%, 0.1% and 0.5% *v*/*v*) and for different durations (0, 5, 10 and 20 min), while for the control (0%), distilled water was used. To assist in the dilution of EO in water, 0.1% *v*/*v* Tween 20 was added. After the application, the fruits were removed from the solution and left to dry in air for 1 h at room temperature. Each treatment consisted of four fruits (i.e., four replications), which were placed in a 5 L polypropylene (PP) plastic container. The containers were placed in an experimental refrigerator at 11 °C and 90% relative humidity (RH) (achieved by placing wet paper inside each box) for appropriate durations of 9 and 12 days for cucumber and tomato, respectively.

#### 2.2.1. Effects on Weight Loss and Respiration Rate

During storage, the fruits’ weight loss and respiration rate were monitored, and sensory evaluation (aroma, appearance and marketability) was also performed for each commodity after an appropriate duration (tomato: days 0, 6 and 12; cucumber: days 0, 6 and 9). Fruit weight was recorded on each of the aforementioned days and the percentage of total weight loss was calculated for each day. The effects on each commodity’s respiration rate were determined as previously described by Xylia et al. [20]. Briefly, the produced carbon dioxide (CO_2_) was measured after hermetically enclosing each fruit at room temperature (RT) in a 1 L plastic container. The air from the container was sucked for 40 s using a dual gas analyzer (GCS 250 Analyzer, International Control Analyser Ltd., Kent, UK). After recording the weight and volume of each fruit, the results were expressed as mL of CO_2_ produced per kg per h (mL CO_2_/kg/h).

#### 2.2.2. Effects on Sensory Attributes

For the sensory evaluation, at least six panelists were employed to assess the aroma, appearance and marketability of the fruits on appropriate days (tomato: days 0, 6 and 12; cucumber: days 0, 6 and 9) [20]. Aroma evaluation was implemented using a 10-point scale (1 interval), which was adapted to the commodity. For tomato, 1: not tomato-like and very unpleasant aroma, 3: not tomato-like and slightly unpleasant aroma, 5: not tomato-like but pleasant aroma, 8: less tomato-like aroma and 10: intense tomato-like aroma. For cucumber, 1: not cucumber-like and very unpleasant aroma, 3: not cucumber-like and slightly unpleasant aroma, 5: not cucumber-like but pleasant aroma, 8: less cucumber-like aroma and 10: intense cucumber-like aroma. Appearance (visual quality and color) was assessed using a 10-point scale (1 interval), which was adapted to the commodity. For tomato, 1: green color of 50%; 3: yellow-green; 5: orange; 8: red; and 10: deep red. For cucumber, 1: yellow color of 50%; 3: yellow-green; 5: light green; 8: green; 10: deep green. For both commodities, marketability (indicating overall quality) was evaluated with the use of a scale of 1–10 (1 interval), where 1: not marketable quality (i.e., malformation, wounds, infection); 3: low marketability with malformation; 5: marketable with few defects, i.e., small size, decolorization (medium quality); 8: marketable (good quality); 10: marketable with no defects (extra quality).

#### 2.2.3. Effects on Quality Parameters

On the initial (day 0) and last days of storage (days 9 and 12 for cucumber and tomato, respectively) quality parameters were also assessed. Fruit firmness was measured at two points on the shoulder of each fruit, using a texture analyzer (TA.XT plus, Stable Micro Systems, Surrey, UK) equipped with a probe 3 mm in diameter travelling at a speed of 2 mm/s and with a penetration depth of 12 mm [20]. The amount of force required to break the fruits’ radial pericarp was recorded in Newtons (N).

The fruits’ surface color was evaluated by recording the L* (brightness/lightness; 0: black/100: white), a* (−a*: greenness and +a*: redness) and b* (−b*: blueness and +b*: yellowness) values (CIELAB uniform color space) with the use of a colorimeter (Chroma meter CR400 Konica Minolta, Tokyo, Japan). Hue (h) was determined in degrees (°) using the following equations: h = 180 + tan^−1^(b*/a*), if a* < 0 and h = tan^−1^(b*/a*), if a* > 0 and b* ≥ 0 (for cucumber and tomato, respectively) [33,34]. The chroma value (C) was calculated as C = (a*2 + b*2)^1/2^ [33]. The color index (CI) was estimated as CI = (a* × 1000)/(L* × b*) [34]. The browning index (BI) was calculated as BI = 100 × (X − 0.31)/0.17, where: X = (a* + 1.75 × L*)/(5.645 × L* + a* − 3.012 × b*) [35]. The yellowing index (YI) was calculated as YI = (142.86 × b*)/L* [35].

For the determination of total soluble solids (TSS), titratable acidity (TA) and sweetness (ripening index), tomato and cucumber tissue were milled using a domestic blender, and then, pressed in a cheesecloth to extract the fruits’ juice. TSS were estimated with the use of a digital portable refractometer (Atago, Tokyo, Japan), while the results were expressed in °Brix. The fruits’ TA was determined via titration with 0.1 N NaOH using the method previously mentioned [36], and results were expressed as g of citric acid for tomatoes (or malic acid for cucumbers) per L of juice. The ratio of TSS over TA (TSS/TA) was used for estimation of the fruits’ sweetness (ripening index).

#### 2.2.4. Effects on Phenolic Content, Antioxidant Activity, Ascorbic Acid and Carotenoid Content

The procedure for the extraction of polyphenols and antioxidants was performed with 50% methanol (*v*/*v*), as previously described by Chrysargyris et al. [37]. The phenolic content of the methanolic extracts was determined via the Folin–Ciocalteu method, as mentioned by Chrysargyris et al. [32]. Briefly, an appropriate volume of methanolic extract was mixed with Folin–Ciocalteu reagent and sodium carbonate 7% (distilled water was added until final volume of 3 mL). After mixing, the reaction was left for 90 min in the dark. The absorbance was measured at 755 nm using a spectrophotometer (Multiskan GO, Thermo Fisher Scientific Oy, Vantaa, Finland) and results were expressed as μg gallic acid (Scharlab, Sentmenat, Spain) equivalents (GAE) per gram of fresh weight (μg GAE/g).

The antioxidant capacity of the fruit extracts was assessed using two different methods: 2,2-diphenyl-1-picrylhydrazyl (DPPH) and ferric-reducing antioxidant power (FRAP) assays. For the DPPH assay, the procedure was performed as described by Chrysargyris et al. [37], whereby an appropriate volume of plant extract was mixed with 0.3 mM DPPH purple solution (Sigma-Aldrich, Taufkirchen, Germany). The reaction solution was mixed and incubated in the dark for 30 min, followed by reading the reaction’s absorbance at 517 nm. The FRAP assay was performed by mixing an appropriate volume of the methanolic extract with 0.3 mL sodium acetate (pH 3.6) (Merck, Darmstadt, Germany), 10 mM tripyridyl-s-triazine (TPTZ) (Sigma-Aldrich, Germany) and 10 mM iron (III) chloride (Scharlau, Spain). The reaction’s absorbance was measured at 593 nm, after incubation at 37 °C [37]. For both assays, the results were quantified using a standard curve of 6-hydroxy-2,5,7,8-tetramethylchroman-2-carboxylic acid (trolox) [(±)-6-hydroxy-2,5,7,8-tetramethylchromane-2-carboxylic acid] (Sigma-Aldrich, Germany) and expressed as μg of trolox per gram of fresh weight (μg trolox/g).

The ascorbic acid (AA) content of tomato and cucumber fruits was determined using the 2,6-dichloroindophenol (DCPIP) titrimetric method, as previously mentioned [36], whereby 10 mL of homogenized, diluted juice (1:10 *v*/*v* diluted in 0.1% oxalic acid) was titrated against standardized DCPIP solution. The results were expressed as mg of ascorbic acid per 100 g of fresh weight (mg AA/100 g).

For tomato fruit, carotenoid content (i.e., *lycopene* and *β-carotene*) was also extracted and estimated using the procedure reported by Nagata and Yamashita [38]. Briefly, one gram (1 g) of blended tomatoes was homogenized with acetone:hexane (4:6, *v*/*v*) and the absorbance of the upper phase formed after the extraction was read at 453, 505, 645 and 663 nm. The results were quantified using the following equations [38]:

*Lycopene* (mg/100 mL *of extract*) = −0.0458 × *A*_663_ + 0.204 × *A*_645_ + 0.372 × *A*_505_ − 0.0806 × *A*_453_ *β-carotene* (mg/100 mL *of extract*) = 0.216 × *A*_663_ − 1.22 × *A*_645_ − 0.304 × *A*_505_ + 0.452 × *A*_453_ (1)

The results were further expressed as mg of carotenoids (*lycopene* and *β-carotene*) per 100 g of fresh weight (mg/100 g).

### 2.3. Assessment of Sanitation Means 

#### 2.3.1. Inoculum Preparation

Bacterial strains of *Salmonella enterica* subsp. *enterica* (ATCC 51741) and *Listeria monocytogenes* (ATCC 19111) were obtained from the Department of Nursing, Cyprus University of Technology. Fresh bacterial cultures (8 log cfu/mL) were prepared in brain–heart infusion broth (BHI broth, HiMedia, Mumbai, India) after overnight (16–18 h) incubation at 37 °C [32].

#### 2.3.2. Procedure

The selected doses (concentration duration of application) from the preliminary screening were further investigated for their ability to lower and/or eliminate the populations of *S. enterica* and *L. monocytogenes* inoculated on tomato and cucumbers fruits. Thus, the following treatments were assessed: (i) sterile distilled water (control), (ii) chlorine (0.02%), (iii) EO Dose A (0.01% for 10 min), (iv) EO Dose B (0.1% for 10 min), (v) hydrosol Dose A (0.1% for 20 min) and (vi) hydrosol Dose B (0.5% for 20 min).

The procedures of inoculation and treatment application were performed as previously described [39], with modifications. The fruits were washed with 0.05% chlorinated water (NaOCl), rinsed with sterile water, and then, air-dried in a laminar flow cabinet. Each fruit was then placed in a labeled sterile stomacher bag. Afterwards, 2 mL of inoculum was sprinkled evenly on each fruit surface (8 log cfu/mL) and bags were left open for 1 h, allowing bacterial attachment on the fruits’ surface. An appropriate volume of the treatment solution (i.e., 50 mL) was added into the bags, and the bags were closed for an appropriate duration. Then, the treatment solution was discarded and the bags were closed and stored at 11 °C for one week. Sampling and microbiological analysis were performed after one and seven days.

#### 2.3.3. Microbiological Analysis

For the determination of the remaining (surviving) inoculum, an appropriate volume of maximum recovery diluent (MRD; Merck, Darmstadt, Germany) was added into each bag (in a 1:10 ratio) [13]. The bags were shaken and the serial decimal dilutions were prepared. From each dilution, 100 μL was spread into the appropriate medium: xylose-lysine-deoxycholate agar (XLD agar; Merck, Darmstadt, Germany) and PALCAM agar (Merck, Darmstadt, Germany) for *S. enterica* and *L. monocytogenes*, respectively. The plates were then incubated at 37 °C for 24–48 h, typical colonies for each bacterium were counted and the results were expressed as log cfu/mL.

### 2.4. Statistical Analysis

The experimental setup was a Completely Randomized Design (CRD) with four biological replications in each treatment. IBM SPSS version 25.0 was used, wherein the data were subjected to one-way analysis of variance (one-way ANOVA) comparing the treatment on each day by performing Duncan’s multiple range test (*p* = 0.05). A paired-samples *t*-Test was also used for comparing control data on the initial and final days.

## 3. Results

### 3.1. Effects on Weight Loss and Respiration Rate

**Figure 1:**
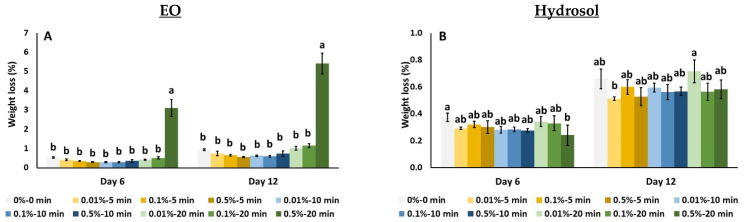

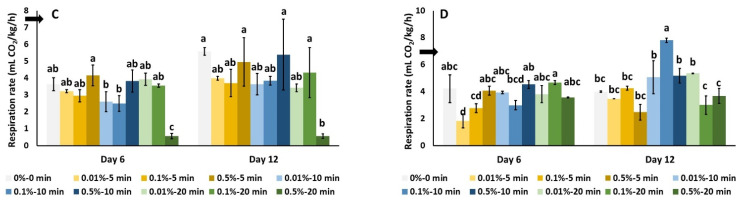
Screening of *O. dubium* EO (**A**,**C**) and hydrosol (**B**,**D**) application effects on weight loss and respiration rate of tomato fruit stored at 11 °C for 12 days. Bars represent the mean ± standard error of four biological replicates per treatment. Measurements for day 0 refer to the control and are indicated with an arrow. On each day, significant differences (*p* < 0.05) among treatments are indicated with different Latin letters.

The effects of *O. dubium* EO and hydrosol application on the weight loss and respiration rate of tomato fruit are illustrated in Figure 1. Increased weight loss was observed with EO at 0.5% for 20 min during the sixth day of storage and this was also evident on the last day of storage (up to 3.11% and 5.42%, respectively) (Figure 1A). The application of *O. dubium* hydrosol at 0.5% for 20 min resulted in lower weight loss compared to non-treated (control) fruit after six days of storage at 11 °C (Figure 1B). However, on the last day of storage (day 12), a decrease in weight loss was observed with the application of hydrosol at 0.01% for 5 min as opposed to at 0.01% for 20 min (0.51% and 0.72%, respectively) (Figure 1B).

The respiration rate of tomato fruit at 6 days was found to increase with the application of EO at 0.5% for 5 min (4.15 mL CO_2_/kg/h) compared to 0.01% for 10 min, 0.1% for 10 min and 0.5% for 20 min (which showed the lowest value even on the 12th day—up to 0.58 mL CO_2_/kg/h) (Figure 1C). The application of hydrosol at 0.1% for 20 min and 0.5% for 10 min, lead to increased respiration rates (4.68 and 4.54 mL CO_2_/kg/h, respectively) compared to 0.01% for 5 min and 0.1% for 5 min (1.84 and 2.78 mL CO_2_/kg/h, respectively) on the sixth day (Figure 1D). On the other hand, the application of hydrosol at 0.1% for 20 min and 0.5% for 20 min was found to decrease the respiration rate compared to 0.01% for 20 min and 10 min (0.01%, 0.1% and 0.5%) (Figure 1D).

**Figure 2:**
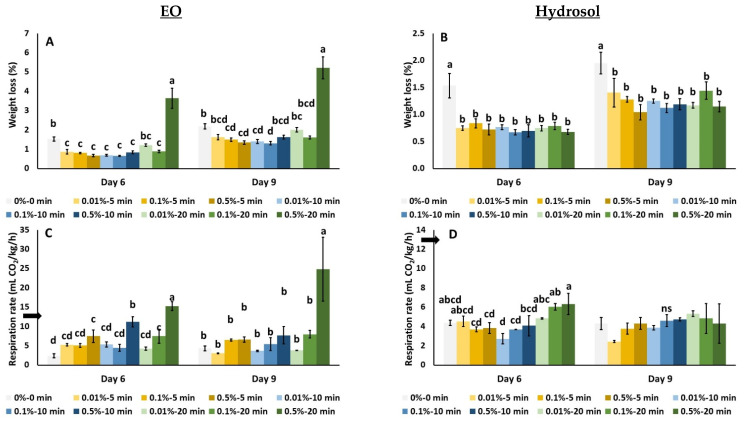
Screening of *O. dubium* EO (**A**,**C**) and hydrosol (**B**,**D**) application effects on weight loss and respiration rate of cucumber fruit stored at 11 °C for 9 days. Bars represent the mean ± standard error of four biological replicates per treatment. Measurements for day 0 refer to the control and are indicated with an arrow. On each day, significant differences (*p* < 0.05) among treatments are indicated with different Latin letters.

Figure 2 presents the effects of *O. dubium* EO and hydrosol application on cucumber’s % weight loss and respiration rate. The application of EO at 0.5% for 20 min presented the greatest weight loss on the sixth day (Figure 2A). On the ninth day, EO at 0.5% for 20 min resulted in significantly increased weight loss, whereas a decrease was observed at 0.1% for 10 min (5.22% and 1.31%, respectively) (Figure 2A). When *O. dubium* hydrosol was applied, a decrease in weight loss was observed with all the applied treatments (compared to the control), on both sampling days (Figure 2B).

As shown in Figure 2C, cucumber’s respiration rate was increased when EO was applied at 0.1% for 20 min and 0.5% for 20 min, 0.5% for 10 min and 0.5% for 5 min compared to the control on the sixth day, and this effect was preserved at 0.5% for 20 min (24.86 mL CO_2_/kg/h) on the ninth day (Figure 2C). The application of *O. dubium* hydrosol resulted in an increased respiration rate at 0.5% for 20 min and 0.1% for 20 min compared to 0.5% for 5 min, 0.1% for 10 min, 0.1% for 5 min and 0.01% for 10 min, after six days of storage at 11 °C (Figure 2D). No significant differences were found between treated and untreated fruit on the ninth day (Figure 2D).

### 3.2. Effects on Sensory Attributes

**Figure 3:**
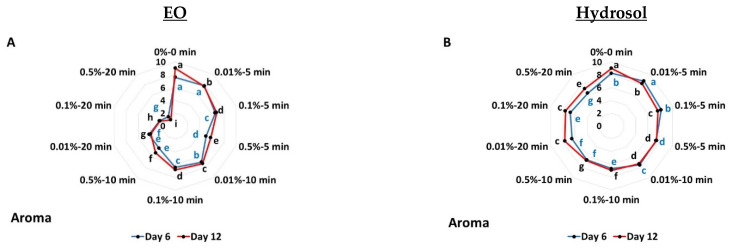

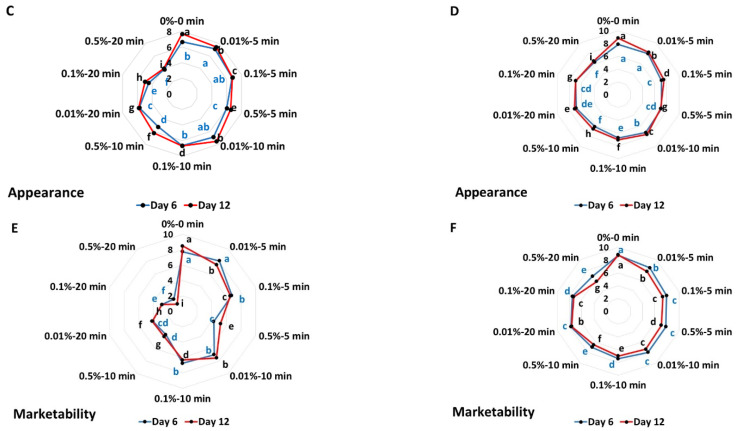
Screening of *O. dubium* EO (**A**,**C**,**E**) and hydrosol (**B**,**D**,**F**) application effects on sensory attributes (aroma, appearance and marketability) of tomato fruit stored at 11 °C for 12 days. Different Latin letters indicate significant differences at *p* < 0.05.

The effects of the applied EO and hydrosol treatments on the sensory attributes (aroma, appearance and marketability) of tomato fruit are shown in Figure 3. During aroma evaluation on the sixth day of storage, decreased scores were recorded for all the applied treatments (except 0.01% for 5 min), while a great decrease in scoring values was reported at 0.5% for 10 min and 0.5% for 20 min (up to 1.79—not tomato-like and very unpleasant aroma) (Figure 3A). During the last day of storage, all applied treatments were found to score lower values on the evaluation scale (Figure 3A). This was more evident as the concentration and the duration were increased. Hydrosol application resulted in decreased scores (except with 0.01% for 5 min; 8.67—less tomato-like aroma), while they did not differ from 0.1% for 5 min on the sixth day (Figure 3B). Moreover, all applied hydrosol treatments presented decreased scores on the last day (Figure 3B).

Tomato’s appearance was found to present lower scores with the application of EO at 0.5% for 5 min, and 0.5% for 10 min and 20 min (0.01%, 0.1% and 0.5%), while higher scores were found with 0.01% for 5 min (7.13—orange-red) (Figure 3C). The application of 0.1% for 5 min, 0.01% for 10 min and 0.1% for 10 min did not differ from the control on the sixth day. After 12 days of storage, all applied EO treatments showed lower values, with greater decreases at 0.5% for 10 min and 20 min (0.01%, 0.1% and 0.5%) (up to 4.00–yellow-orange) (Figure 3C). When hydrosol was applied, a lower score was reported on day six (up to 6.21—orange-red) except 0.01% for 5 min, while 0.5% for 10 min and 0.5% for 20 min did not differ between them (Figure 3D). All applied hydrosol treatments were shown to present lower scores on the last day (day 12) (Figure 3D).

Essential oil-treated tomatoes revealed that on the last day of storage, all applied treatments resulted in decreased scores, and this was more evident as the concentration and duration of application were increased (Figure 3E and Figure S1). All applied hydrosol treatments were found to result in decreased marketability, whilst no significant differences were observed between 0.1% for 5 min, 0.5% for 5 min, 0.01% for 10 min and 0.01% for 20 min, on the sixth day (Figure 3F).

**Figure 4:**
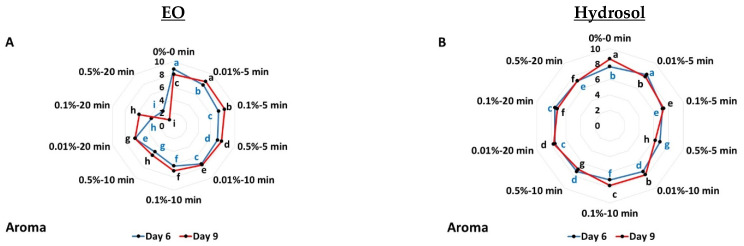

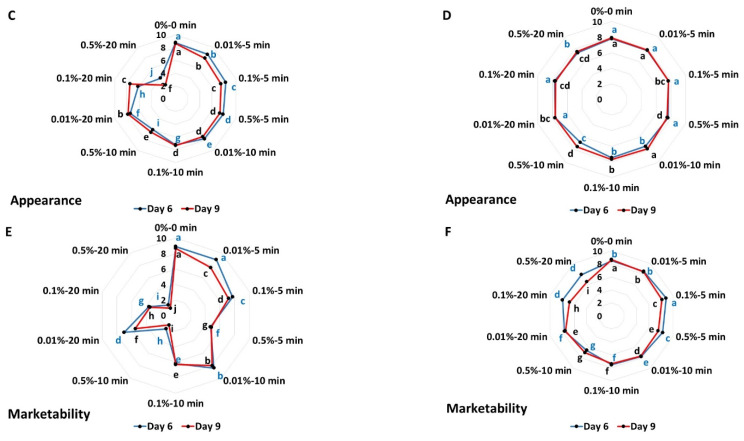
Screening of *O. dubium* EO (**A**,**C**,**E**) and hydrosol (**B**,**D**,**F**) application effects on sensory attributes: aroma (aroma, appearance and marketability) of cucumber fruit stored at 11 °C for 9 days. Different Latin letters indicate significant differences at *p* < 0.05.

Figure 4 presents the effects of *O. dubium* EO and hydrosol application on cucumber’s sensory attributes (aroma, appearance and marketability). All applied EO treatments resulted in decreased aroma scoring values on the sixth day, and this was evident at 9 days of storage (except for applications at 0.01% for 5 min and 0.1% for 5 min) (Figure 4A). When hydrosol was applied, a decrease in scoring was observed on the sixth day for all applied treatments, whilst treatment at 0.01% for 5 min presented increased score (7.83—less cucumber-like aroma) (Figure 4B).

The appearance of cucumber was reported to present lower scores with the EO applications and especially at 0.5% for 10 and 0.5% for 20 min (6.00–light green and 4.00–yellow-green, respectively) on the sixth day (Figure 4C). Decreased scores were also observed on the last day (day 9), especially at 0.5% for 20 min, whereas no significant differences were observed between 0.5% for 5 min, 0.01% for 10 min, 0.1% for 10 min and 0.01% for 20 min (Figure 4C). Cucumber’s appearance was reported to present low scores with hydrosol application for 10 min (0.01%, 0.1% and 0.5%) and at 0.5% for 20 min on the sixth and ninth days (Figure 4D).

Decreased marketability scores were reported on the sixth day with EO application (except 0.01% for 5 min; 8.92—marketable (good quality)), while on the last day, all EO treatments presented lower values, especially at 0.5% for 5 min, 0.5% for 10 min, 0.1% for 20 min and 0.5% for 20 min (Figure 4E and Figure S2). Hydrosol application presented low scores (except 0.01% for 5 min that did not differ from the control) on day six (Figure 4F). All applied hydrosol treatments resulted in decreased scoring values on the last day with a much greater decrease on hydrosol at 0.5% (5, 10 and 20 min) (Figure 4F).

### 3.3. Effects on Quality Attributes

The effects of EO or hydrosol on tomato fruit firmness, TSS, TA and sweetness after storage for 12 days at 11 °C are presented in Table 1. The application of EO for 10 min (0.01%, 0.1% and 0.5%) and at 0.01% for 20 min maintained firmness compared to 0.1% for 20 min and 0.5% for 20 min after 12 days of storage. The application of *O. dubium* hydrosol resulted in lower firmness in fruit treated with 0.5% for 5 min compared to 0.01% for 20 min (8.53 and 12.86 N, respectively). Fruit firmness decreased in control samples during storage. Hydrosol application increased tomato’s TSS on day 12 at 0.5% for 20 min (3.90 °Brix) compared to 0.5% for 10 min and 0.01% for 5 min (3.30 and 3.27 °Brix, respectively) (Table 1).

Titratable acidity was not affected by EO use 12 days after application (Table 1). The application of hydrosol for 10 and 20 min (0.01%, 0.1% and 0.5%) revealed low TA of tomato fruit on the last day of storage compared to the non-treated (control) fruit. The ratio of TSS/TA (i.e., sweetness) was increased at 0.1% for 20 min compared to 0.1% for 10 min (1.38 and 0.94, respectively) (Table 1). When hydrosol was applied for 20 min (0.01%, 0.1% and 0.5%) and at 0.5% for 10 min, increased sweetness was observed compared to the control, 0.01% for 5 min and 0.5% for 5 min. 

Table 2 shows the effects of *O. dubium* EO and hydrosol application on the firmness, TSS, TA and sweetness of cucumber fruit stored at 11 °C for 9 days. When EO was applied at 0.1% for 20 min, 0.5% for 5 min, 0.5% for 10 min and 0.5% for 20 min, it maintained fruit firmness (19.01, 18.90, 16.52 and 16.38 N, respectively) compared to the control (12.68 N). Interestingly, the application of EO for 10 min (0.01%, 0.1% and 0.5%), as well as at 0.01% for 20 min and 0.5% for 20 min, decreased the TSS of cucumbers at the end of storage (day 9), whilst hydrosol application did not significantly affect cucumber’s TSS (Table 2).

As shown in Table 2, a decrease in cucumber’s TA was reported with 20 min EO application (0.01%, 0.1% and 0.5%) compared to the control. In contrast, hydrosol application at 0.5% for 20 min revealed increased cucumber TA compared to 0.01% for 5 min (1.23 and 0.72 g malic acid/L, respectively) (Table 2). Cucumber’s sweetness was found to be increased when treated with EO at 0.1% for 20 min and 0.5% for 20 min compared to the control, and at 0.01% for 5 min and 0.01% for 10 min (Table 2). When hydrosol was applied at 0.01% for 5 min and 0.1% for 10 min, it resulted in increased cucumber sweetness, whereas treatment at 0.5% for 20 min did not significantly differ from the control.

The effects of *O. dubium* EO and hydrosol application on tomato’s color are presented in Table 3. The application of EO did not significantly affect the L* values of the treated fruit, whereas hydrosol application at 0.5% for 10 min, 0.01% for 20 min and 0.1% for 20 min increased the L* value, unlike 0.5% for 5 min and 0.5% for 20 min. A decrease in the a* value was observed with the application of 0.5% for 20 min compared to the control. When hydrosol at 0.5% for 5 min was applied, a decrease in tomato’s a* value was reported compared to the control. Comparing the control on the initial and last day, an increased a* value was reported during the latter. The b* value was found to be increased with EO at 0.1% for 20 min compared to 0.5% for 20 min, 0.01% for 10 min and 0.01% for 5 min (Table 3). On the other hand, hydrosol at 0.5% for 5 min, 0.1% for 10 min and 0.5% for 20 min decreased the b* values compared to the control. Comparing the control, on the initial and last day, an increased b* value was reported during the latter.

Hue value was increased with the application of EO at 0.5% for 20 min compared to all the other applied treatments (except 0.1% for 20 min), whereas hydrosol application did not significantly affect tomato’s hue value (Table 3). Chroma was found to increase with the application of EO at 0.1% for 20 min compared to all the applied treatments on the last day (day 12). In contrast, hydrosol application at 0.1% for 5 min, 0.5% for 5 min and 0.1% for 10 min resulted in decreased chroma values compared to the control (Table 3). An increase in chroma value was reported for the control on day 12 when compared with day 0 (initial day). As shown in Table 3, the color index was increased with EO application at 0.5% for 20 min compared to 0.01% for 20 min, 0.5% for 10 min, 5 min (0.01%, 0.1% and 0.5%) and the control. Hydrosol application also resulted in an increased color index at 0.5% for 20 min compared to all applied treatments (except 0.5% for 5 min and 0.01% for 10 min).

The browning index on tomato fruit increased with EO application at 0.1% for 20 min, while a decreased browning index was reported on tomato fruit when treated with hydrosol at 0.1% for 5 min, 0.5% for 5 min, 0.1% for 10 min, 0.5% for 10 min and 0.01% for 20 min compared to the non-treated fruit. Comparing the control on the initial and last days, an increased browning index was reported during the latter. An increased yellowing index was observed on the last day of tomatoes’ storage with EO application at 0.5% for 10 min and 0.1% for 20 min compared to 0.5% for 20 min, 0.01% for 5 min, 0.1% for 5 min and 0.01% for 10 min (Table 3). However, hydrosol application at 0.1% for 5 min, 0.5% for 5 min, 0.5% for 10 min and 0.01% for 20 min resulted in a decreased yellowing index compared to the control. Comparing the control on the initial and the last days, an increase in the yellowing index was observed during the latter.

The cucumber color parameters as affected by the applied treatments of *O. dubium* EO and hydrosol are shown in Table 4. The application of EO at 0.1% for 10 min decreased cucumber’s hue value compared to the control. On the other hand, an increased hue value was observed at 0.1% for 10 min compared to 0.01% for 5 min. All other color parameters measured were not significantly different between the applied treatments.

### 3.4. Effects on Phenolic Content, Antioxidant Activity, Ascorbic Acid and Carotenoid Content

Table 5 shows the effects of O. dubium EO and hydrosol application on tomato’s phenolic content, antioxidant activity, ascorbic acid and carotenoid content (*lycopene* and *β-carotene*). Total phenols were found to increase with the application of EO at 0.5% for 20 min, followed by 0.5% for 5 min, whereas the application of hydrosol at 0.5% for 10 min increased the phenolic content of tomato fruit compared to the other treatments. The antioxidant activity (assessed via DPPH) of tomato increased with EO at 0.01% for 5 min and 0.5% for 10 min compared to all other treatments (except 0.1% for 10 min) (Table 5). The same assay showed that a decrease in antioxidants was caused by hydrosol at 0.01% for 10 min compared to 0.5% for 5 min, 0.5% for 10 min and 0.1% for 20 min. On the other hand, increased antioxidants were reported with hydrosol at 0.5% for 10 min compared to 0.1% for 5 min and 0.01% for 10 min (Table 5). The FRAP assay revealed increased antioxidant activity with EO application at 0.01% for 5 min and 0.5% for 5 min compared to the control, 0.1% for 5 min, and 0.01% for 10 min and 20 min (0.01%, 0.1% and 0.5%). When hydrosol was applied at 0.1% for 10 min and 0.5% for 10 min, an increase in antioxidants was reported compared to 0.1% for 5 min, 0.01% for 10 min and 0.01% for 20 min (Table 5).

As shown in Table 5, tomato’s AA content was increased by EO application at 0.1% for 5 min, 0.5% for 5 min, 0.01% for 10 min, 0.01% for 20 min and 0.1% for 20 min compared to non-treated fruit, whereas 0.5% for 20 min did not significantly differ from the control. An increase in AA was also observed for all applied hydrosol treatments. *Lycopene* was found to increase with EO at 0.1% for 20 min (2.16 mg *Lycopene*/100 g) compared to 0.01% for 20 min, 0.5% for 20 min, 0.01% for 10 min, 0.5% for 10 min and 0.5% for 5 min (1.44, 1.26, 1.48, 1.54 and 1.05 mg *Lycopene*/100 g) (Table 5). Hydrosol at 0.01% for 5 min and 0.5% for 5 min decreased *Lycopene* content compared to all applied treatments (0.1% for 5 min and 0.1% for 10 min). EO application at 0.01% for 10 min and 0.1% for 20 min resulted in increased *β-carotene* content compared to all other treatments (except 0.1% for 5 min, 0.1% for 10 min). Hydrosol increased *β-carotene* content when applied at 0.5% for 5 min, 0.5% for 10 min and 0.01% for 20 min. Comparing the control on the initial and last days, an increase in carotenoids (*Lycopene* and *β-carotene*) was observed during the latter (Table 5). 

The effects of *O. dubium* EO and hydrosol application on cucumber’s phenolic content, antioxidant activity (DPPH, FRAP) and ascorbic acid content are presented in Table 6. Total phenolic content was decreased with the application of *O. dubium* EO at 0.1% for 20 min, 0.1% for 10 min and 0.5% for 10 min compared to the control and 0.01% for 5 min. When hydrosol was applied, decreased phenolic content was obtained at 0.5% for 10 min and 0.5% for 5 min compared to the control. The DPPH assay showed that EO application at 0.5% for 20 min increased tomato’s antioxidants compared to all other treatments (Table 6). According to the results from the FRAP assay, an increase in antioxidants was reported with EO at 0.5% for 20 min, whereas 0.1% for 10, 0.5% for 10 and 0.5% for 20 min resulted in decreased antioxidants compared to the control and 5 min (0.01%, 0.1% and 0.5%). Hydrosol application presented a decrease in cucumber’s antioxidants (at 0.01% for 5 min and 0.01% for 20 min). Cucumber’s AA content was found to increase with EO at 0.5% for 20 min compared to the control, 0.01% for 5 min and 0.5% for 5 min (Table 6). Moreover, the AA content of cucumber fruit stored at 11 °C for 9 days was increased by hydrosol at 0.5% for 10 min and 0.01% for 5 min compared to the control.

### 3.5. Assessment of Sanitation Means

**Figure 5:**
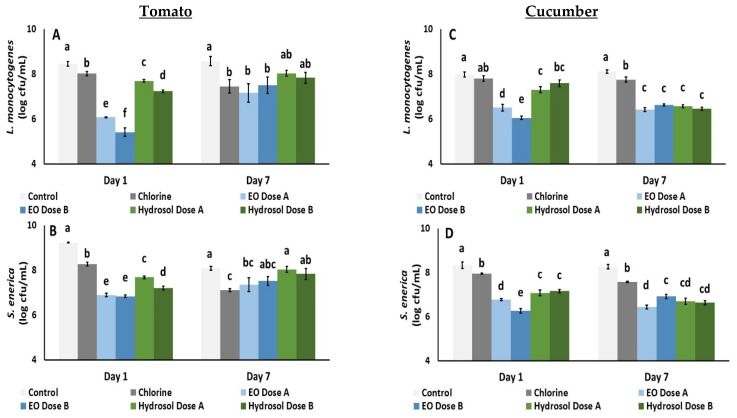
Effects of sanitation means, i.e., chlorine (0.02%), *O. dubium* EO dose A (0.01% for 10 min), EO dose B (0.1% for 10 min), hydrosol dose A (0.1% for 20 min) and hydrosol dose B (0.5% for 20 min) on tomato (**A**,**B**) and cucumber (**C**,**D**) fruits stored 11 °C and inoculated with *Listeria monocytogenes* and *Salmonella enterica*, respectively. Bars represent the mean ± standard error of four biological replicates per treatment. On each day, significant differences (*p* < 0.05) among treatments are indicated with different Latin letters.

The in vivo antibacterial activity against *Listeria monocytogenes* and *Salmonella enterica* in the selected applied doses of *O. dubium* EO and hydrosol on tomato and cucumber fruits is illustrated on Figure 5. All applied treatments were found to decrease the *L. monocytogenes* population on tomato fruit; there was a great decrease resulting from EO dose B (0.1% for 10 min), followed by dose A (0.01% for 10 min), with a 3.04 and 2.38 log decrease, respectively, on the first day of storage. The population of *S. enterica* inoculated on tomato fruit was decreased by all applied treatments on the first day of storage, with a greater decrease being reported with EO. A decrease in *S. enterica* was observed with chlorine and EO dose A on the last day (day 7) (0.97 and 0.73 log decrease, respectively) (Figure 5).

The population of *L. monocytogenes* was decreased on cucumbers with all the applied treatments (except chlorine) on the first day of storage, and all treatments were able to decrease the bacterial population on the last day (day 7) (Figure 5). However, no significant differences were reported between EO and hydrosol doses. *S. enterica* numbers were decreased with all applied treatments on the first day. This decrease was more evident with EO dose B, followed by EO dose A (2.07 and 1.57 log decrease, respectively), while no significant difference was observed between hydrosol doses. On the seventh day of storage, the *S. enterica* population was decreased with all applied treatments (especially with EO dose A—up to a 1.84 log decrease) (Figure 5). Interestingly, no significant differences were found between EO dose B and hydrosol (both doses) on the same day.

## 4. Discussion

Tomatoes and cucumbers are among the most widely consumed fresh produce. However, during storage, great losses might occur due to mechanical damage and deterioration (decay), among others. Weight loss due to water loss (via the respiration and transpiration processes) can result in senescing, browning, a loss in fruit texture and flavor, susceptibility to chilling injury and membrane disintegration, among others [40,41]. In the present study, both fruits presented a great weight loss percentage (up to 5.22%) with the application of EO at 0.5% for 20 min, whereas hydrosol application resulted in a decreased percentage of weight loss (up to 1.40%). In a previous study, the application of 0.5% sage EO on tomato fruit for 10 min resulted in increased weight loss compared to 0.1% and non-treated fruits, even after 14 days of storage at 11 °C [42]. These findings might be attributed to the increased concentration and longer duration of application of the EOs, as well as the hydrophilic nature of the hydrosol (colloidal suspensions of essential oil and water-soluble components) which could possibly hydrate the fruit surface, preventing water loss.

Climacteric fruits are characterized by increased ethylene production and respiration rates during their ripening stage [21]. Tomato, as a climacteric fruit, exhibits these increases. It seems, from the present study, that different commodities responded differently to the same treatment; this might be due to the fact that tomato is a climacteric fruit, whereas cucumber is a non-climacteric fruit, having different metabolic responses, different maturation stages (green- and red-colored fruits) and timeframes. A previous study by Tzortzakis et al. [42] showed that the application of 0.5% sage EO increased respiration in tomato fruit after 7 and 14 days. In another study, the application of an eco-product based on EOs from eucalyptus and rosemary (at concentrations of 0.4 and 0.8%) did not significantly affect the respiration rate and ethylene production of cucumber fruit, even after 14 days of storage at 11 °C [43]. The reported differences in the respiration rate could be attributed to the cell wall disruption and disturbance of gas exchange caused by the EO application [24,25]. In the present study, the decrease in respiration rate found in tomato fruit might be the result of severe stress due to the high concentration (0.5%) combined with longer duration of application (20 min). Moreover, any shifts in tomato’s respiration, as a climacteric fruit, could have taken place prior to the sixth day of storage, and thus, may not have been recorded.

Among the most important factors shaping consumers’ purchasing decisions, optical/visual quality (i.e., appearance) and aroma are noticed first. The application of EOs should be carried out with caution as they are products with intense and distinct aromas that could interfere with the fresh produce’s own aroma, resulting in an unpleasant final product. In the present study, it was observed that tomato treated with EO at increased concentrations and for longer durations presented an unpleasant aroma, whereas hydrosol application resulted in a less tomato-like (but acceptable) aroma. Moreover, increased EO and hydrosol levels and longer durations of application caused tissue burning/spotting for both tomato and cucumber fruit (Figures S1 and S2). Tzortzakis et al. [42] reported positive effects (i.e., greater appearance, aroma, texture and marketability) on tomato fruit treated with sage EO (0.1%) compared to non-treated ones. However, when a higher concentration of the same sage EO (0.5%) was used, a less acceptable product was observed [42], as was the case in the present work. It seems that cucumber is more sensitive to EO in comparison to tomato, probably due to the different fruit surface and/or secondary metabolism (Figure S2). Notably, at the end of storage, the strong aroma of the applied EO faded out. However, EO applied at high concentrations revealed an unpleased odor in the examined fresh produce. When cucumbers were treated with an eco-product (containing rosemary and eucalyptus EOs) at a concentration of 0.8%, they presented a less intense cucumber-like aroma compared to the non-treated ones [43]. The same study mentioned no differences in cucumber’s appearance [43]. When investigating the effects of EO and other natural product applications on fresh produce and other food, one might have in mind the volatile nature of these products, since they tend to lose their essence when reaching the end of the fresh produce storage period. Fruits that have longer storage life will possibly be affected less by EO vapors at the end of the storage period. Furthermore, an appropriate combination of EO and fresh produce in which they complement each other should be considered too.

The vibrant color of fruits increases consumers’ interest in purchasing. In our case, EO (especially at 0.01% for 5 min) and hydrosol application resulted in more orange-red colored tomato fruits and greener cucumbers. Cucumber fruit treated with an eco-product based on eucalyptus and rosemary EOs (via the dipping method) was found not to affect fruit’s appearance (at concentrations of 0.4 and 0.8%) [43]. The findings of the present study could be attributed to the antioxidant activity of EOs and hydrosols, which could possibly prevent the oxidation of essential fruit pigments (i.e., *Lycopene*, *β-carotene* and chlorophylls) as well as prevent undesirable enzymatic activities (i.e., enzymatic browning) [20,26]. A previous study has shown that dipping tomato fruit in 0.4% of an eco-product (containing eucalyptus and rosemary EOs) resulted in decreased marketability after seven days of storage [20]. In another study, more marketable tomato fruits were reported with the application of 0.1% sage EO compared to 0.5% EO treatment and the control, even after 14 days of application and storage at 11 °C [42]. The variance in the effects among the studies might be due to the different EOs used, their composition and the conditions of application (concentration and duration of exposure), among others. When EO concentration and the duration of application increased, nonmarketable tomato and cucumber fruits were observed. Adams et al. [44] reported that the application of ginger EO for 20 and 30 min resulted in more acceptable tomato fruit compared to the no treatment. The differences between the effects of *O. dubium* EO and hydrosol might have been caused by the EO’s “stronger” chemical profile since EOs tend to be more active than the respective hydrosols [20,23].

Consumers, when purchasing fresh produce, tend to prefer more firm fruits (i.e., cucumbers and tomatoes) as they have longer fridge and/or shelf lives. In the present study, firmness was maintained in tomato fruit treated with EO applications at 0.01%, 0.1% and 0.5% for 10 min, 0.5% for 5 min and 0.01% for 20 min. Maintenance of fruit firmness was also observed with the application of 0.1% sage EO, while a higher concentration (0.5%) was found to result in softer fruits [42]. The maintenance of fruit firmness might be attributed to the antioxidant capacity of EOs and hydrosols, which can reduce free radicals and protect fruits’ cell walls from oxidation and subsequent degradation [45,46]. These findings are also supported by another study wherein cherry tomatoes were treated with oregano (*Origanum vulgare*) (up to 3%) incorporated in packaging film, which maintained firmness even after a week of storage at 4 and 22 °C [47].

The sweetness of fruits progress during the maturation stage, where fruits’ acids are reduced and sugars are increased. During fruit maturation, the TSS increase and the TA decrease is related to the conversion of starch and acids into sugars [48]. Previous research has shown a decrease in TSS in fruits (tomato and apple) treated with EOs, especially when combined with edible coatings [44,49], which practically shows a delay in fruit ripening/maturation. Decreased values of TA suggest a delay in the maturation of tomato fruits. Sweeter tomato fruits were noted with the application of EO at 0.1% for 20 min compared to 0.1% for 10 min. It has been previously mentioned that tomatoes treated with EOs were found to present decreased TSS as a result of their respiration rate [21]. Thus, it seems that this statement confirms the findings of our study, especially in the longer duration of applications (10 and 20 min). It is noteworthy that tomato fruits at different maturation stages can be affected differently when dipped in EOs. For instance Chrysargyris et al. [17] found that tomatoes at the mature stage (red colored) presented increased TSS, whereas tomatoes at the breaker stage (green colored) presented low TSS values. In the same study, the authors did not observe any significant differences in the TA of tomato fruits at both maturation stages (breaker and mature) [17]. Taking into account that cucumber fruits are harvested and consumed when they are in the breaker stage (immature fruit) might explain our observations of lower TSS values during EO application.

As with many fruits and vegetables, tomatoes and cucumbers are an important source of phytonutrients such as antioxidants. In our study, the phenolic content of tomatoes increased with EO at 0.5% for 20 min and 0.5% for 5 min, as well as with hydrosol at 0.5% for 10 min. On the other hand, cucumber’s phenolic content was found to decrease with EO at 0.1% for 20 min, 0.1% for 10 min and 0.5% for 10 min, as well as with the application of hydrosol at 0.5% for 10 min and 0.5% for 5 min. The antioxidant activity of tomato fruit was found to increase with EO at 0.01% for 5 min. Similarly, an increase in antioxidants was observed in tomatoes treated with hydrosol at 0.5% for 10 min compared to the ones treated with 0.1% for 5 min and 0.01% for 10 min. For cucumbers, antioxidants were found to increase with EO at 0.5% for 20 min, whereas hydrosol application resulted in decreased antioxidants. It has been previously shown that EO application in fruits and vegetables can ignite the mechanisms related to plant tissue defense such as the production of antioxidants and the activity of antioxidant enzymes [50]. This confirms the findings of the present study and further suggests that *O. dubium* EO and hydrosol application in fresh produce (i.e., tomato and cucumbers) could result in products with increased nutritional value (i.e., increased polyphenols, antioxidants and ascorbic acid). However, these observations seem to vary due to different concentration–time combinations. Based on the literature, the antioxidant capacity of oregano extracts is due to both non-phenolic compounds (acting as scavengers of free radicals) and phenolic compounds (disrupting the chain of processes that consume oxygen using a mechanism similar to how tocopherols work) [51]. The antioxidant properties of *O. dubium* EO are associated with carvacrol content, which is the main EO component [52].

The increase in fruits’ (for both commodities) AA and tomato’s carotenoid (*Lycopene* and *β-carotene*) content observed in the present study might be attributed to the antioxidant activity of EOs (and other plant extracts such as hydrosols), which can ignite the production of antioxidants in fruits as well as prevent the oxidation and loss of components of high nutritional value (i.e., antioxidants and vitamins) [50,53]. From the observations of the present study, these suggest an increase in the nutritional value of fresh produce. These compounds play a major role in plant protection against oxidative processes that can damage essential cell components and, at the same time, are beneficial for human health, acting against oxidative stress and chronic illnesses (i.e., cancer, chronic inflammation, etc.) [54,55].

The essential oil of *O. dubium* has been shown to present great in vitro antioxidant and antimicrobial activity; however, there is no known information about its in vivo application in fresh produce or its effects [4]. In the present study, EO (0.1% for 10 min) was found to be more effective between the applied treatments (chlorine, EO and hydrosol) in both fruits. The results observed in the present study might be attributed to the ability of the pathogen to attach itself to fruit surfaces, the morphology of the fruit surfaces, and the volatile nature and composition of the EO. Several EOs from different species, such as eucalyptus lemon, helichrysum, sage, nutmeg, cinnamon and clove, inhibited the growth of *E. coli* in cucumber fruit, preserving the fruit’s quality and flavor [56]. Oregano EO-treated tomatoes revealed decreased fruit decay after 14 days of storage [57]. A previous study showed that EO from *Satureja thymbra* presented great antibacterial activity against *Staphylococcus simulans*, *Lactobacillus fermentum*, *Pseudomonas putida*, *S. enterica* and *L. monocytogenes* [58,59]. The main components of *S. thymbra* EO are γ-terpinene and carvacrol, which are responsible for the antibacterial activity of this EO, and are also the main compounds of *O. dubium*. It seems that these compounds might present a synergistic effect in the action of EOs against a plethora of bacterial pathogens and non-pathogens. Moreover, *O. dubium* EO and hydrosol were found to be more effective against *L. monocytogenes* (in both fruits) compared to *S. enterica*. This is due to the different bacterial cell wall structure of Gram-bacteria (i.e., *S. enterica*) which contains a double lipid layer preventing hydrophobic molecules (i.e., components of EOs) from entering the cell wall [38]. Similarly, Basil et al. [60] found Gram+ bacteria (*Clavibacter michiganensis* subsp. *michiganensis*) to be more sensitive than three Gram- bacteria, including *Pseudomonas syringae* pv. *tomato*, *Xanthomonas axonopodis* pv. *Vesicatoria* and *Xanthomonas axonopodis* pv. *phaseoli*, when exposed to *O. dubium* EO. The fungicidal, insecticidal, antimicrobial and anticarcinogenic properties of *O. dubium* EO are related to the increased levels of carvacrol content [52]. A stronger antibacterial effect could be obtained if the fruit was vaporized rather than dipped in the EO, as it has been previously reported that the volatile antibacterial activity of *O. dubium* EO was more effective than contact antibacterial activities [60].

The exploitation of MAPs for obtaining EOs and other plant extracts, and their use during postharvest treatments of fresh produce, should take place in moderation, especially for endemic species, and they should only be used as long as they can be cultivated and present high EO yields and quality. Another thing to consider for the use of essential oils is that they are considered to be a product of high cost; however, using them in the optimum dose (concentration × duration of application) might be cost-effective. Another factor for consideration when using EOs is the possible phytotoxicity when applied to produce and allergy issues during consumption. On the other hand, hydrosols are considered as by-products produced in greater quantities (opposed to EOs), with their biological activity providing the opportunity for further investigation of their uses.

## 5. Conclusions

To conclude, Cypriot oregano (*O. dubium*) EO and hydrosol could be considered as alternative sanitizing agents of tomato and cucumber fruits. The applied EO doses (0.01% for 10 min and 0.1% for 10 min) were able to decrease the population of two main foodborne pathogens (*L. monocytogenes* and *S. enterica*—up to a 3.04 log decrease for *L. monocytogenes*). Similarly, *O. dubium* hydrosol (0.1% for 20 min and 0.5% for 20 min) resulted in decreased bacterial numbers. The most efficient sanitizing agent among the applied treatments (chlorine, EO and hydrosol) in the present study was found to be EO application at 0.1% for 10 min (in both fruits). The lower concentrations combined with shorter durations of application (i.e., EO: 0.01% for 10 min and hydrosol: 0.5% for 10 min) were found to preserve the quality attributes (i.e., aroma and appearance) of tomato and cucumber, increasing the antioxidants and phenolic content of the produce and resulting in products of slightly increased nutritional value. Based on these promising results, further research is needed on *O. dubium* product (EOs, hydrosol and extracts) application at different doses and on other fresh produce.

## Figures and Tables

**Table 1:** Screening of *O. dubium* EO and hydrosol application effects on fresh tomato firmness, total soluble solids (TSS), titratable acidity (TA) and sweetness when stored at 11 °C for 12 days. Values presented as mean ± standard error of four biological replicates per treatment. Measurements for day 0 refer to the control. In each column and for each treatment (EO and hydrosol), significant differences (*p* < 0.05) are indicated with different Latin letters. For control (0.00%), the asterisk (*) indicates the difference (*p* < 0.05) on the initial and final days (day 0 and day 12, respectively).

	Time (min)	Concentration	Firmness (N)	TSS (°Brix)	TA (Citric Acid g/L)	Sweetness (TSS/TA)
Day 0	0	0.00%	15.47 ± 1.16	3.40 ± 0.07	3.70 ± 0.01	0.92 ± 0.03
EO	0	0.00%	10.75 ± 0.68 ab *	3.60 ± 0.06	3.83 ± 0.20	0.94 ± 0.04 ab
	5	0.01%	10.55 ± 0.61 ab	3.60 ± 0.00	3.91 ± 0.57	0.95 ± 0.12 ab
		0.1%	10.22 ± 0.77 ab	3.57 ± 0.09	3.64 ± 0.22	0.99 ± 0.09 ab
		0.5%	12.86 ± 0.74 a	3.53 ± 0.03	4.04 ± 0.15	0.88 ± 0.03 ab
	10	0.01%	11.77 ± 0.85 a	3.67 ± 0.07	3.88 ± 0.28	0.95 ± 0.05 ab
		0.1%	12.83 ± 0.75 a	3.33 ± 0.09	4.40 ± 0.34	0.77 ± 0.08 b
		0.5%	12.58 ± 0.83 a	3.53 ± 0.23	3.96 ± 0.49	0.91 ± 0.09 ab
	20	0.01%	12.58 ± 1.38 a	3.60 ± 0.10	3.89 ± 0.23	0.93 ± 0.05 ab
		0.1%	7.92 ± 1.70 bc	3.57 ± 0.07	3.55 ± 0.17	1.01 ± 0.05 a
		0.5%	5.56 ± 1.15 c	3.47 ± 0.09	3.66 ± 0.10	0.95 ± 0.01 ab
Hydrosol	0	0.00%	10.75 ± 0.68 ab *	3.60 ± 0.06 ab	3.83 ± 0.20 a	0.94 ± 0.04 d
	5	0.01%	11.17 ± 1.16 ab	3.27 ± 0.23 b	3.46 ± 0.17 abc	0.94 ± 0.02 d
		0.1%	10.34 ± 0.67 ab	3.40 ± 0.06 ab	3.52 ± 0.04 abc	0.97 ± 0.01 cd
		0.5%	8.53 ± 0.39 b	3.43 ± 0.12 ab	3.72 ± 0.18 ab	0.92 ± 0.03 d
	10	0.01%	11.00 ± 0.91 ab	3.40 ± 0.15 ab	3.15 ± 0.04 cd	1.08 ± 0.04 bcd
		0.1%	10.22 ± 0.34 ab	3.50 ± 0.17 ab	3.26 ± 0.18 bcd	1.08 ± 0.04 bcd
		0.5%	10.67 ± 1.29 ab	3.30 ± 0.15 b	2.88 ± 0.10 d	1.15 ± 0.08 b
	20	0.01%	12.86 ± 1.87 a	3.37 ± 0.13 ab	2.99 ± 0.10 cd	1.13 ± 0.02 bc
		0.1%	11.54 ± 1.06 ab	3.80 ± 0.31 ab	2.90 ± 0.29 d	1.31 ± 0.05 a
		0.5%	10.07 ± 0.28 ab	3.90 ± 0.21 a	2.87 ± 0.17 d	1.38 ± 0.11 a

**Table 2:** Screening of *O. dubium* EO and hydrosol application effects on fresh cucumber firmness, total soluble solids (TSS), titratable acidity (TA) and sweetness when stored at 11 °C for 9 days. Values presented as mean ± standard error of four biological replicates per treatment. Measurements for day 0 refer to the control. In each column and for each treatment (EO and hydrosol), significant differences (*p* < 0.05) are indicated with different Latin letters. For control (0.00%), the asterisk (*) indicates the difference (*p* < 0.05) on the initial and final days (day 0 and day 9, respectively).

	Time (min)	Concentration	Firmness (N)	TSS (°Brix)	TA (Malic Acid g/L)	Sweetness (TSS/TA)
Day 0	0	0.00%	14.71 ± 1.48	2.93 ± 0.09	0.99 ± 0.02	2.95 ± 0.06
EO	0	0.00%	12.68 ± 0.63 c	3.30 ± 0.21 a	1.11 ± 0.02 a *	2.97 ± 0.22 c
	5	0.01%	13.78 ± 1.02 bc	2.90 ± 0.10 abc	0.97 ± 0.05 ab	3.01 ± 0.14 c
		0.1%	15.31 ± 0.88 bc	3.03 ± 0.67 ab	0.85 ± 0.08 abc	3.62 ± 0.31 bc
		0.5%	18.90 ± 0.83 a	2.93 ± 0.15 abc	0.77 ± 0.01 abc	3.83 ± 0.19 bc
	10	0.01%	13.87 ± 1.08 bc	2.83 ± 0.07 bc	0.85 ± 0.04 abc	3.35 ± 0.20 c
		0.1%	13.87 ± 1.28 bc	2.83 ± 0.07 bc	0.81 ± 0.00 abc	3.48 ± 0.08 bc
		0.5%	16.52 ± 1.22 ab	2.53 ± 0.12 c	0.90 ± 0.44 abc	4.29 ± 1.49 bc
	20	0.01%	15.50 ± 0.64 bc	2.83 ± 0.15 bc	0.61 ± 0.05 bc	4.72 ± 0.16 abc
		0.1%	19.01 ± 0.59 a	2.93 ± 0.13 abc	0.57 ± 0.05 bc	5.22 ± 0.36 ab
		0.5%	16.38 ± 1.19 ab	2.77 ± 0.18 bc	0.44 ± 0.02 c	6.37 ± 0.60 a
Hydrosol	0	0.00%	12.68 ± 0.63 b	3.30 ± 0.21	1.11 ± 0.02 ab *	2.97 ± 0.22 b
	5	0.01%	13.96 ± 1.04 ab	3.20 ± 0.17	0.72 ± 0.10 b	4.58 ± 0.52 a
		0.1%	14.00 ± 1.03 ab	3.57 ± 0.12	0.92 ± 0.07 ab	3.92 ± 0.35 ab
		0.5%	14.74 ± 1.30 ab	3.43 ± 0.22	0.84 ± 0.01 ab	4.07 ± 0.25 ab
	10	0.01%	16.04 ± 1.13 ab	3.50 ± 0.21	0.87 ± 0.04 ab	4.03 ± 0.15 ab
		0.1%	14.83 ± 0.83 ab	3.70 ± 0.12	0.86 ± 0.01 ab	4.33 ± 0.12 a
		0.5%	15.54 ± 1.19 ab	3.33 ± 0.07	0.89 ± 0.03 ab	3.76 ± 0.21 ab
	20	0.01%	16.96 ± 0.94 a	3.37 ± 0.09	0.91 ± 0.03 ab	3.70 ± 0.23 ab
		0.1%	15.50 ± 1.03 ab	3.43 ± 0.19	0.85 ± 0.00 ab	4.04 ± 0.21 ab
		0.5%	17.28 ± 1.26 a	3.30 ± 0.00	1.23 ± 0.35 a	3.08 ± 0.70 b

**Table 3:** Screening of *O. dubium* EO and hydrosol application effects on fresh tomato color parameters (L*, a*, b*, hue (h), chroma value (C), color index (CI), browning index (BI) and yellowing index (YI)) when stored at 11 °C for 12 days. Values presented as mean ± standard error of four biological replicates per treatment. Measurements for day 0 refer to the control. In each column and for each treatment (EO and hydrosol), significant differences (*p* < 0.05) are indicated with different Latin letters. For control (0.00%), the asterisk (*) indicates the difference (*p* < 0.05) on the initial and final days (day 0 and day 12, respectively).

	Time (min)	Concentration	L*	a*	b*	H	C	CI	ΒΙ	YI
Day 0	0	0.00%	49.13 ± 1.35	12.36 ± 1.57	21.70 ± 0.16	60.62 ± 3.17	25.09 ± 0.73	11.76 ± 1.90	75.86 ± 4.66	63.23 ± 1.50
EO	0	0.00%	45.54 ± 0.48	20.45 ± 0.67 ab *	29.22 ± 0.87 b *	55.00 ± 1.20 b	35.68 ± 0.80 bc *	15.42 ± 0.73 a	128.90 ± 6.12 ab *	91.73 ± 3.45 ab *
	5	0.01%	45.14 ± 0.65	20.41 ± 0.48 ab	27.42 ± 1.36 b	53.24 ± 1.00 b	34.20 ± 1.31 bc	16.58 ± 0.65 a	121.99 ± 8.60 b	86.90 ± 5.01 b
		0.1%	44.09 ± 0.20	19.31 ± 1.51 ab	26.04 ± 1.73 b	53.49 ± 0.71 b	32.43 ± 2.27 bc	16.80 ± 0.37 a	117.34 ± 10.97 b	84.38 ± 5.71 b
		0.5%	45.36 ± 1.44	20.44 ± 1.11 ab	31.05 ± 2.35 ab	56.46 ± 1.52 b	37.21 ± 2.41 abc	14.78 ± 1.28 a	139.21 ± 11.10 ab	97.61 ± 6.04 ab
	10	0.01%	46.45 ± 0.72	18.41 ± 1.04 b	28.16 ± 0.67 b	56.91 ± 0.90 b	33.65 ± 1.12 bc	14.05 ± 0.54 ab	117.33 ± 5.09 b	86.65 ± 2.35 b
		0.1%	46.64 ± 0.30	22.38 ± 0.95 a	34.95 ± 1.21 ab	57.38 ± 0.22 b	41.50 ± 1.53 ab	13.73 ± 0.15 ab	157.12 ± 8.13 ab	107.05 ± 3.62 ab
		0.5%	45.24 ± 0.90	22.64 ± 0.92 a	32.23 ± 0.77 ab	54.94 ± 1.18 b	39.41 ± 0.90 abc	15.60 ± 0.98 a	149.15 ± 7.09 ab	101.90 ± 3.14 a
	20	0.01%	46.45 ± 1.12	21.64 ± 0.97 ab	30.92 ± 2.59 ab	54.74 ± 2.02 b	37.81 ± 2.42 abc	15.42 ± 1.54 a	135.49 ± 10.04 ab	94.77 ± 5.97 ab
		0.1%	46.74 ± 1.17	21.36 ± 0.67 ab	39.89 ± 8.55 a	59.53 ± 4.72 ab	45.80 ± 7.52 a	12.87 ± 2.28 ab	244.06 ± 109.62 a	122.93 ± 28.38 a
		0.5%	46.22 ± 0.46	13.56 ± 2.30 c	27.52 ± 1.78 b	64.35 ± 3.06 a	30.81 ± 2.44 c	10.53 ± 1.48 b	108.33 ± 11.09 b	85.11 ± 5.61 b
Hydrosol	0	0.00%	45.54 ± 0.48 abc	20.45 ± 0.67 a *	29.22 ± 0.87 a *	55.00 ± 1.20	35.68 ± 0.80 a *	15.42 ± 0.73 ab	128.90 ± 6.12 a *	91.73 ± 3.45 a *
	5	0.01%	46.12 ± 0.61 ab	17.98 ± 1.46 ab	27.34 ± 1.02 ab	56.83 ± 1.69	32.76 ± 1.51 abc	14.24 ± 0.93 b	113.83 ± 4.94 ab	84.64 ± 2.19 ab
		0.1%	46.18 ± 0.72 ab	17.23 ± 0.13 ab	25.92 ± 0.37 abc	56.38 ± 0.33	31.13 ± 0.34 bc	14.42 ± 0.33 b	106.30 ± 1.34 b	80.21 ± 0.87 b
		0.5%	43.78 ± 0.73 c	16.19 ± 0.63 b	23.73 ± 0.93 c	55.67 ± 1.34	28.75 ± 0.90 c	15.68 ± 1.02 ab	102.30 ± 3.73 b	77.41 ± 2.40 b
	10	0.01%	44.73 ± 0.44 bc	18.41 ± 0.06 ab	27.18 ± 0.16 ab	55.89 ± 0.20	32.83 ± 0.13 abc	15.15 ± 0.21 ab	118.58 ± 1.78 ab	86.84 ± 1.01 ab
		0.1%	44.73 ± 0.77 bc	16.87 ± 2.10 ab	25.52 ± 1.67 bc	56.93 ± 1.58	30.62 ± 2.55 bc	14.58 ± 0.69 b	108.42 ± 9.32 b	81.37 ± 4.49 b
		0.5%	47.40 ± 0.20 a	18.98 ± 1.02 ab	26.64 ± 0.64 abc	54.60 ± 1.17	32.73 ± 1.00 abc	15.03 ± 0.71 ab	108.36 ± 4.18 b	80.30 ± 2.08 b
	20	0.01%	47.75 ± 0.53 a	19.78 ± 0.30 ab	26.87 ± 1.00 abc	53.56 ± 1.42	33.40 ± 0.63 ab	15.63 ± 0.95 ab	110.35 ± 2.96 b	80.87 ± 2.43 b
		0.1%	46.16 ± 0.63 ab	18.31 ± 1.67 ab	26.99 ± 1.59 abc	56.02 ± 1.09	32.63 ± 2.23 abc	14.61 ± 0.45 b	112.70 ± 7.82 ab	83.42 ± 3.40 ab
		0.5%	43.94 ± 0.92 c	18.98 ± 0.93 ab	25.38 ± 0.69 bc	53.27 ± 0.70	31.70 ± 1.09 abc	17.04 ± 0.77 a	114.28 ± 7.28 ab	82.74 ± 3.78 ab

**Table 4:** Screening of *O. dubium* EO and hydrosol application effects on fresh cucumber color parameters (L*, a*, b*, hue (h), chroma value (C), color index (CI), browning index (BI) and yellowing index (YI)) when stored at 11 °C for 9 days. Values presented as mean ± standard error of four biological replicates per treatment. Measurements for day 0 refer to the control. In each column and for each treatment (EO and hydrosol), significant differences (*p* < 0.05) are indicated with different Latin letters.

	Time (min)	Concentration	L*	a*	b*	H	C	CI	ΒΙ	YI
Day 0	0	0.00%	35.86 ± 1.23	−11.75 ± 0.43	15.02 ± 0.68	128.06 ± 0.48	19.07 ± 0.79	−21.95 ± 1.05	23.91 ± 0.92	59.82 ± 1.51
EO	0	0.00%	40.79 ± 2.26	−14.02 ± 0.99	20.15 ± 2.08	125.06 ± 0.93 a	24.56 ± 2.27	−17.47 ± 1.49	34.31 ± 3.83	70.07 ± 3.61
	5	0.01%	42.27 ± 2.95	−14.64 ± 1.47	22.33 ± 3.51	123.98 ± 1.75 ab	26.73 ± 3.73	−16.51 ± 2.43	39.73 ± 7.62	74.02 ± 7.04
		0.1%	38.93 ± 3.20	−11.99 ± 2.39	17.48 ± 3.98	124.96 ± 1.13 a	21.21 ± 4.63	−18.49 ± 2.10	30.07 ± 7.28	61.83 ± 9.50
		0.5%	40.43 ± 2.17	−12.70 ± 1.51	19.09 ± 2.94	124.08 ± 1.17 ab	22.94 ± 3.27	−17.02 ± 1.60	33.55 ± 5.98	66.37 ± 6.91
	10	0.01%	43.45 ± 3.19	−15.83 ± 1.16	24.36 ± 3.07	123.55 ± 1.63 ab	29.09 ± 3.19	−15.81 ± 2.25	43.74 ± 5.96	79.10 ± 4.75
		0.1%	43.01 ± 2.40	−13.27 ± 3.02	23.74 ± 1.59	117.80 ± 5.17 b	27.46 ± 2.61	−12.31 ± 2.19	48.78 ± 4.67	78.70 ± 0.93
		0.5%	44.08 ± 3.13	−14.65 ± 1.69	23.69 ± 3.54	122.22 ± 1.16 ab	27.87 ± 3.89	−14.72 ± 1.87	42.77 ± 6.59	75.36 ± 6.79
	20	0.01%	40.34 ± 2.57	−13.57 ± 1.72	19.60 ± 3.90	125.75 ± 2.06 a	23.88 ± 4.18	−18.36 ± 2.29	34.19 ± 9.37	67.95 ± 10.03
		0.1%	41.03 ± 2.15	−14.25 ± 1.09	20.83 ± 2.29	124.66 ± 1.06 ab	25.25 ± 2.49	−17.12 ± 1.58	36.55 ± 5.35	71.99 ± 5.12
		0.5%	39.90 ± 1.28	−13.14 ± 0.60	19.83 ± 1.50	123.77 ± 1.81 ab	23.83 ± 1.43	−16.96 ± 1.52	36.47 ± 4.53	70.84 ± 3.94
Hydrosol	0	0.00%	40.79 ± 2.26	−14.02 ± 0.99	20.15 ± 2.08	125.06 ± 0.93 ab	24.56 ± 2.27	−17.47 ± 1.49	34.31 ± 3.83	70.07 ± 3.61
	5	0.01%	43.44 ± 1.77	−14.96 ± 0.66	22.71 ± 1.31	123.47 ± 0.89 b	27.20 ± 1.41	−15.37 ± 1.19	39.37 ± 2.07	74.54 ± 1.90
		0.1%	40.54 ± 1.49	−13.68 ± 1.10	19.45 ± 2.23	125.40 ± 1.06 ab	23.79 ± 2.45	−17.68 ± 1.23	32.87 ± 5.18	68.03 ± 5.59
		0.5%	41.06 ± 2.00	−14.33 ± 1.40	20.96 ± 2.85	124.75 ± 1.07 ab	25.40 ± 3.14	−17.14 ± 1.52	36.91 ± 6.55	72.06 ± 6.73
	10	0.01%	42.17 ± 0.58	−15.19 ± 0.36	22.77 ± 1.32	123.85 ± 0.98 ab	27.38 ± 1.29	−15.96 ± 0.79	41.22 ± 4.16	77.05 ± 3.62
		0.1%	39.02 ± 0.63	−13.20 ± 0.48	17.67 ± 0.81	126.79 ± 0.39 a	22.06 ± 0.93	−19.19 ± 0.50	28.32 ± 1.80	64.64 ± 2.25
		0.5%	43.41 ± 1.45	−15.97 ± 0.84	23.88 ± 2.05	123.98 ± 1.01 ab	28.74 ± 2.16	−15.65 ± 1.06	42.37 ± 5.20	78.31 ± 4.84
	20	0.01%	42.30 ± 1.06	−15.26 ± 0.79	21.94 ± 1.91	125.07 ± 1.10 ab	26.74 ± 2.01	−16.70 ± 1.11	37.41 ± 5.22	73.82 ± 5.18
		0.1%	40.62 ± 1.79	−14.00 ± 0.86	19.62 ± 2.04	125.83 ± 1.19 ab	24.11 ± 2.16	−18.01 ± 1.56	32.37 ± 4.32	68.45 ± 4.22
		0.5%	39.31 ± 2.54	−14.31 ± 0.88	19.49 ± 1.57	126.41 ± 0.68 ab	24.18 ± 1.78	−19.08 ± 1.57	32.90 ± 2.75	70.77 ± 3.37

**Table 5:** Screening of *O. dubium* EO and hydrosol application effects on fresh tomato phenols, antioxidants (DPPH, FRAP), ascorbic acid (AA) and carotenoid (*Lycopene*, *β-carotene*) content when stored at 11 °C for 12 days. Values presented as mean ± standard error of four biological replicates per treatment. Measurements for day 0 refer to the control. In each column and for each treatment (EO and hydrosol), significant differences (*p* < 0.05) are indicated with different Latin letters. For control (0.00%), the asterisk (*) indicates the difference (*p* < 0.05) on the initial and final days (day 0 and day 12, respectively).

	Time (min)	Concentration	Phenols (μg GEA/g)	DPPH (μg trolox/g)	FRAP (μg trolox/g)	AA (mg/100 g)	*Lycopene* (mg/100 g)	*β-carotene* (mg/100 g)
Day 0	0	0.00%	163.68 ± 14.33	291.24 ± 5.62	194.15 ± 19.77	5.52 ± 0.72	0.86 ± 0.03	0.57 ± 0.02
EO	0	0.00%	168.51 ± 9.90 c	257.96 ± 8.97 b	218.79 ± 10.22 bcd	6.23 ± 0.28 b	1.67 ± 0.16 abc *	0.66 ± 0.03 de *
	5	0.01%	208.29 ± 8.02 bc	314.96 ± 7.70 a	276.39 ± 34.33 a	8.80 ± 1.08 ab	2.03 ± 0.23 ab	0.71 ± 0.04 cd
		0.1%	156.67 ± 5.21 c	238.51 ± 12.11 bcd	174.67 ± 10.47 d	9.73 ± 0.69 a	1.75 ± 0.14 abc	0.93 ± 0.09 ab
		0.5%	237.14 ± 8.13 b	262.11 ± 12.99 b	279.95 ± 15.29 a	10.28 ± 1.04 a	1.05 ± 0.17 d	0.60 ± 0.13 de
	10	0.01%	161.81 ± 5.43 c	255.10 ± 18.77 b	198.88 ± 9.52 cd	9.41 ± 0.78 a	1.48 ± 0.06 cd	0.98 ± 0.03 a
		0.1%	187.29 ± 3.17 bc	274.08 ± 11.96 ab	244.75 ± 10.46 abc	8.35 ± 1.40 ab	1.67 ± 0.14 abc	0.88 ± 0.06 abc
		0.5%	191.03 ± 17.37 bc	315.48 ± 24.16 a	263.67 ± 12.23 ab	9.07 ± 1.21 ab	1.54 ± 0.21 bcd	0.75 ± 0.03 bcd
	20	0.01%	177.07 ± 7.66 bc	201.95 ± 13.14 d	195.50 ± 4.48 cd	10.22 ± 0.30 a	1.44 ± 0.06 cd	0.73 ± 0.01 cd
		0.1%	196.40 ± 13.16 bc	249.85 ± 9.38 bc	208.65 ± 11.48 cd	9.83 ± 0.76 a	2.16 ± 0.13 a	1.06 ± 0.04 a
		0.5%	339.81 ± 53.32 a	206.98 ± 18.93 cd	184.77 ± 14.50 d	6.46 ± 0.28 b	1.26 ± 0.12 cd	0.50 ± 0.03 e
Hydrosol	0	0.00%	168.51 ± 9.90 cd	257.96 ± 8.97 abc	218.79 ± 10.22 abc	6.23 ± 0.28 c	1.67 ± 0.16 ab *	0.66 ± 0.03 cd *
	5	0.01%	169.67 ± 10.76 cd	264.42 ± 12.57 abc	207.96 ± 30.97 abc	10.98 ± 0.56 b	1.04 ± 0.21 c	0.55 ± 0.09 d
		0.1%	146.17 ± 4.23 d	234.69 ± 7.68 bc	168.04 ± 7.71 bc	12.01 ± 0.58 b	1.29 ± 0.17 bc	0.75 ± 0.08 bcd
		0.5%	194.07 ± 21.96 bc	293.14 ± 31.27 ab	190.51 ± 30.56 abc	11.69 ± 2.47 b	1.99 ± 0.15 a	1.04 ± 0.14 a
	10	0.01%	141.02 ± 8.67 d	225.82 ± 9.29 c	157.82 ± 16.65 c	11.29 ± 0.59 b	1.98 ± 0.04 a	0.97 ± 0.06 ab
		0.1%	220.84 ± 20.86 ab	270.89 ± 17.59 abc	239.26 ± 28.79 a	13.82 ± 0.74 ab	1.21 ± 0.22 bc	0.71 ± 0.07 bcd
		0.5%	245.23 ± 5.80 a	316.21 ± 10.04 a	238.52 ± 8.64 a	12.60 ± 0.77 b	2.11 ± 0.20 a	1.05 ± 0.04 a
	20	0.01%	158.93 ± 8.01 cd	267.38 ± 22.13 abc	160.34 ± 11.68 c	12.09 ± 1.08 b	2.13 ± 0.28 a	1.04 ± 0.12 a
		0.1%	191.31 ± 18.22 bc	288.21 ± 13.83 ab	230.52 ± 12.21 ab	16.01 ± 1.32 a	2.12 ± 0.08 a	0.83 ± 0.05 abc
		0.5%	175.03 ± 13.95 cd	264.50 ± 27.51 abc	177.39 ± 8.94 abc	11.31 ± 0.52 b	1.87 ± 0.17 a	0.97 ± 0.02 ab

**Table 6:** Screening of *O. dubium* EO and hydrosol application effects on fresh cucumber phenols, antioxidants (DPPH, FRAP) and ascorbic acid (AA) content when stored at 11 °C for 9 days. Values presented as mean ± standard error of four biological replicates per treatment. Measurements for day 0 refer to the control. In each column and for each treatment (EO and hydrosol), significant differences (*p* < 0.05) are indicated with different Latin letters.

	Time (min)	Concentration	Phenols (μg GEA/g)	DPPH (μg trolox/g)	FRAP (μg trolox/g)	AA (mg AA/100 g)
Day 0	0	0.00%	62.28 ± 1.74	13.03 ± 1.86	31.56 ± 0.98	5.31 ± 0.45
EO	0	0.00%	69.88 ± 4.23 a	9.54 ± 0.80 de	26.80 ± 1.44 b	3.22 ± 0.86 bc
	5	0.01%	67.67 ± 2.27 a	12.55 ± 0.74 bc	25.94 ± 0.69 b	3.16 ± 0.40 c
		0.1%	60.70 ± 5.58 abc	12.59 ± 1.37 bc	26.06 ± 1.20 b	3.43 ± 0.72 abc
		0.5%	57.16 ± 4.88 abc	11.58 ± 0.99 bcd	24.45 ± 1.02 b	3.10 ± 0.45 c
	10	0.01%	56.90 ± 2.45 abc	10.46 ± 0.52 cde	23.99 ± 0.39 bc	3.33 ± 0.12 abc
		0.1%	53.82 ± 4.51 bc	11.52 ± 0.48 bcd	20.78 ± 0.12 cd	3.77 ± 0.40 abc
		0.5%	53.65 ± 4.87 bc	9.02 ± 0.47 e	18.83 ± 1.56 d	3.50 ± 0.27 abc
	20	0.01%	60.35 ± 2.62 abc	13.52 ± 0.42 b	20.34 ± 1.21 d	3.85 ± 1.08 abc
		0.1%	49.98 ± 2.80 c	11.34 ± 0.24 bcde	17.90 ± 1.39 d	5.17 ± 0.41 ab
		0.5%	66.06 ± 3.79 ab	17.04 ± 0.60 a	39.63 ± 1.63 a	5.22 ± 0.47 a
Hydrosol	0	0.00%	69.88 ± 4.23 a	9.54 ± 0.80 cd	26.80 ± 1.44 a	3.22 ± 0.86 b
	5	0.01%	60.66 ± 4.89 ab	10.28 ± 0.28 bcd	24.26 ± 2.86 ab	5.15 ± 0.78 a
		0.1%	65.16 ± 3.19 ab	9.75 ± 1.16 cd	18.45 ± 1.17 bcd	3.64 ± 0.67 ab
		0.5%	51.43 ± 4.86 b	12.85 ± 0.72 ab	18.67 ± 3.29 bcd	4.55 ± 0.20 ab
	10	0.01%	57.82 ± 4.32 ab	11.14 ± 1.14 abc	17.67 ± 0.99 cd	4.10 ± 0.50 ab
		0.1%	55.19 ± 7.56 ab	7.30 ± 0.80 de	17.32 ± 1.17 cd	4.12 ± 0.24 ab
		0.5%	52.27 ± 4.33 b	6.26 ± 1.08 ef	19.65 ± 0.78 bc	5.07 ± 0.40 a
	20	0.01%	66.47 ± 5.82 ab	7.97 ± 1.11 de	23.04 ± 2.38 abc	4.41 ± 0.31 ab
		0.1%	59.10 ± 2.66 ab	3.90 ± 1.08 f	12.57 ± 1.00 d	4.37 ± 0.47 ab
		0.5%	65.34 ± 3.68 ab	13.49 ± 0.96 a	17.84 ± 2.08 cd	4.80 ± 0.58 ab

## Data Availability

Not applicable.

## Supplementary Materials

The following supporting information can be downloaded at: https://www.mdpi.com/article/10.3390/biology11121772/s1, Figure S1. Screening of *O. dubium* EO and hydrosol application on tomatoes; Figure S2. Screening of *O. dubium* EO and hydrosol application on cucumbers.
