# Supplementary material for: Origanum dubium (Cypriot Oregano) as a Promising Sanitizing Agent against Salmonella enterica and Listeria monocytogenes on Tomato and Cucumber Fruits
Source: Biology (Basel). 2022 Dec 6;11(12):1772. doi: 10.3390/biology11121772 (PMC9775658; doi:10.3390/biology11121772)
Supplement: Supplementary file 1 [file biology-11-01772-s001.zip › biology-2029194-supplementary.pdf]

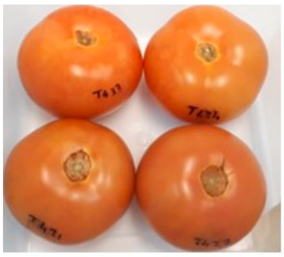

0.5%-20 min

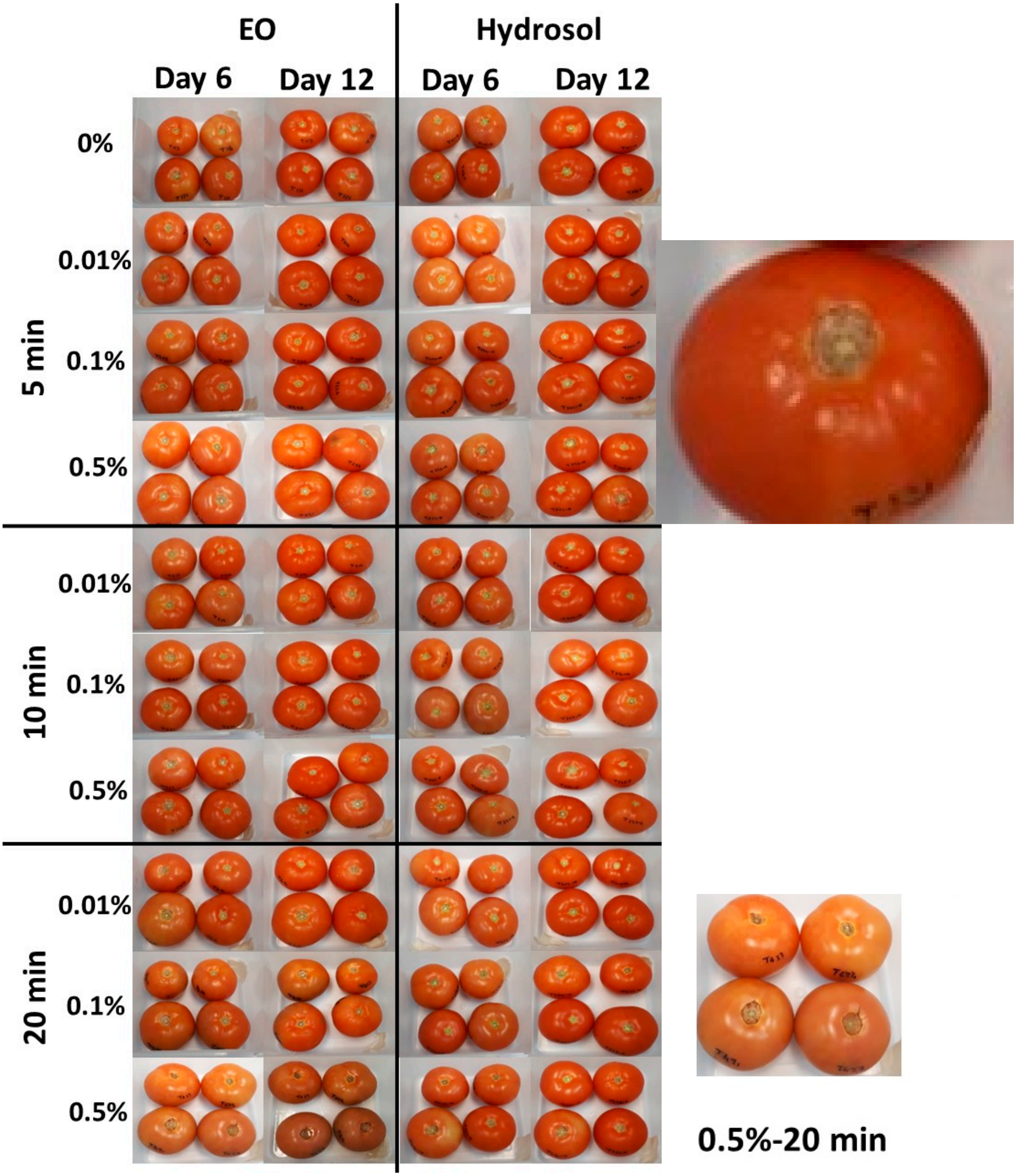

Figure S1. Screening of *O. dubium* EO and hydrosol application on tomatoes

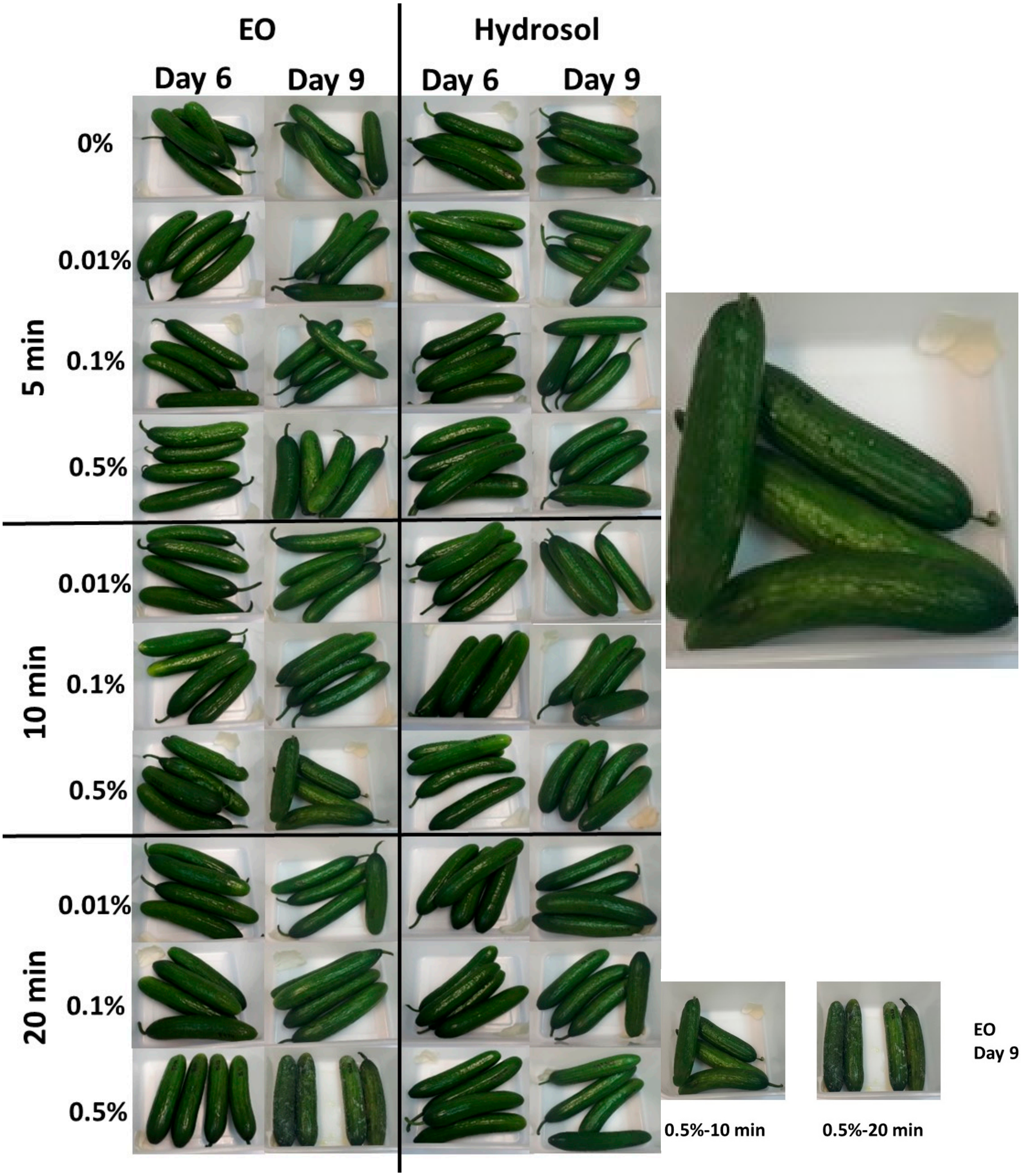

Figure S2. Screening of *O. dubium* EO and hydrosol application on cucumbers.
